# Radiation myelitis after pembrolizumab administration, with favorable clinical evolution and safe rechallenge: a case report and review of the literature

**DOI:** 10.1186/s40425-019-0803-x

**Published:** 2019-11-21

**Authors:** Marcela Carausu, Arnaud Beddok, Adriana Langer, Nicolas Girard, François-Clément Bidard, Marie-Ange Massiani, Damien Ricard, Luc Cabel

**Affiliations:** 1 Department of Medical Oncology, Institut Curie, Saint Cloud, France; 2 Department of Radiotherapy, Institut Curie, Saint Cloud, France; 3 Department of Radiology, Institut Curie, Saint Cloud, France; 4 Department of Medical Oncology, Institut Curie, Paris, France; 5 Université de Lyon, Université Claude Bernard Lyon 1, Lyon, France; 6 Université de Versailles Saint-Quentin-en-Yvelines, Université Paris-Saclay, Paris, France; 7 Department of Neurology, Service de Santé des Armées, Hôpital d’instruction des Armées Percy, Clamart, France; 8 Ecole du Val-de-Grâce, Service de Santé des Armées, Paris, France

**Keywords:** Radiation myelitis, Pembrolizumab, Lung cancer, Immune checkpoint inhibitor

## Abstract

**Background:**

Neurologic complications as myelitis are very rare but extremely deleterious adverse effects of both immunotherapy and radiotherapy. Many recent studies have focused on the possible synergy of these two treatment modalities due to their potential to enhance each other’s immunomodulatory actions, with promising results and a safe tolerance profile.

**Case presentation:**

We report here the case of a 68-year-old man with metastatic non-small-cell lung cancer (NSCLC) who developed myelitis after T12-L2 vertebral radiotherapy, with motor deficit and sphincter dysfunction, while on treatment with pembrolizumab (an immune checkpoint inhibitor). The spinal abnormalities detected by magnetic resonance imaging (MRI), suggestive of myelitis, faithfully matched the area previously irradiated with 30 Gy in 10 fractions, six and a half months earlier. After immunotherapy discontinuation and steroid treatment, the patient rapidly and completely recovered. On progression, pembrolizumab was rechallenged and, after 8 cycles, the patient is on response and there are no signs of myelitis relapse.

**Conclusion:**

The confinement within the radiation field and the latency of appearance are suggestive of delayed radiation myelopathy. Nevertheless, the relatively low dose of radiation received and the full recovery after pembrolizumab discontinuation and steroid therapy plead for the contribution of both radiotherapy and immunotherapy in the causality of this complication, as an enhanced inflammatory reaction on a focal post-radiation chronic inflammatory state. In the three previously described cases of myelopathy occurring after radiotherapy and immunotherapy, a complete recovery had not been obtained and the immunotherapy was not rechallenged. The occurrence of a radiation recall phenomenon, in this case, can not be excluded, and radiation recall myelitis has already been described with chemotherapy and targeted therapy. Safe rechallenges with the incriminated drug, even immunotherapy, have been reported after radiation recall, but we describe it for the first time after myelitis.

## Background

The spinal cord is a critical dose-limiting organ in the context of radiotherapy, with possibly devastating consequences of its radiation-induced toxicity.

Radiation myelopathy can occur in two different clinical patterns. Early delayed or transient myelopathy usually occurs after a delay of 6 weeks to 6 months, mostly consists of Lhermitte’s phenomenon and is self-limiting. Delayed or progressive myelopathy is a chronic progressive disease, usually developing after more than 6 months after the completion of radiotherapy (most often after 9 to 15 months) [1–3]. Its clinical manifestations range from minor motor and sensory deficits to a Brown–Séquard syndrome, transverse myelopathy, and bladder and bowel dysfunctions [1]. There is no proven long-term treatment, although several strategies might bring temporary and partial improvement, such as steroid therapy, hyperbaric oxygen, anticoagulation, or antiangiogenics [1].

Radiation myelopathy is a rare condition, especially with the improvement in the delivery techniques, but reports of it have recently reemerged in the context of spine stereotactic body radiation therapy, or combination therapy with anticancer drugs (chemotherapy, targeted drugs or immunotherapy) [4–6].

The synergistic effects of radiotherapy and immunotherapy as an anticancer association are increasingly being studied, with multiple trials showing promising results [7], but also the possible occurrence of pathologic immune responses and synergistic adverse effects, as well [7, 8].

Pembrolizumab is an immune checkpoint inhibitor (ICI), an anti-PD-1 antibody, approved for the treatment of metastatic non-small-cell lung cancer (NSCLC).

We report here the case of a patient who developed myelopathy while under pembrolizumab for metastatic NSCLC, at six and a half months after he underwent radiotherapy for metastatic spine lesions, with full recovery of the myelopathy and safe rechallenge of the ICI.

## Case presentation

**Fig. 1:**
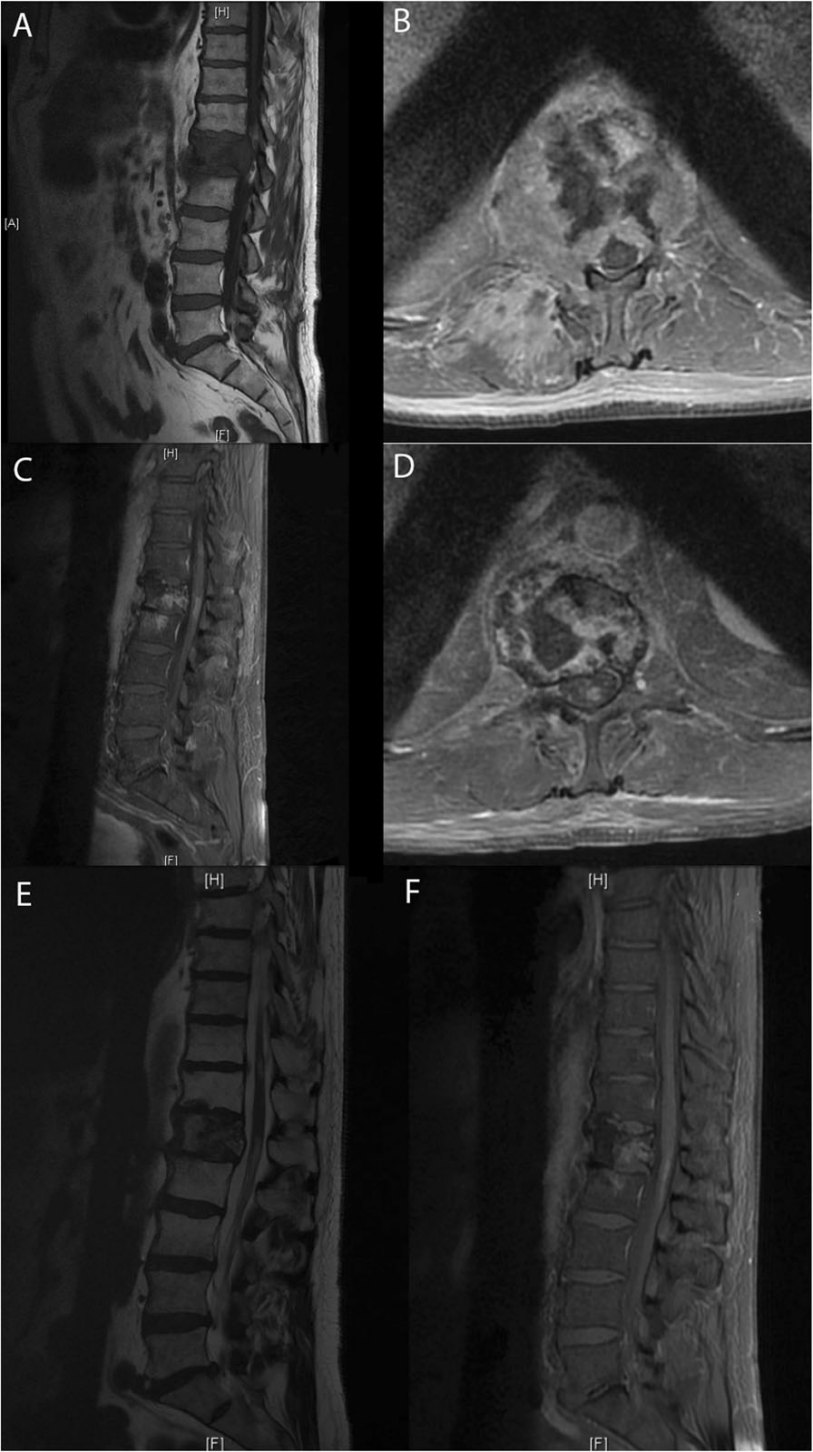
The radiological evolution of myelitis. **a** and **b** MRI performed at the time of epiduritis diagnosis, sagittal T1-weighted spin-echo and axial fat-suppressed T1 after gadolinium injection show osseous metastasis of L1 with epiduritis (but no enhancement of the spinal cord). **c** and **d** MRI after the first signs of myelitis, sagittal and axial fat-suppressed T1 after gadolinium injection show abnormal enhancement of the conus medullaris, and regression of osseous involvement and epiduritis. **e** MRI at 1 month after the discontinuation of immunotherapy, sagittal T2-weighted spin-echo shows hyperintensity of the conus medullaris. **f** MRI at 3.5 months, sagittal fat-suppressed T1 after gadolinium injection shows the persistence of conus medullaris enhancement

**Fig. 2:**
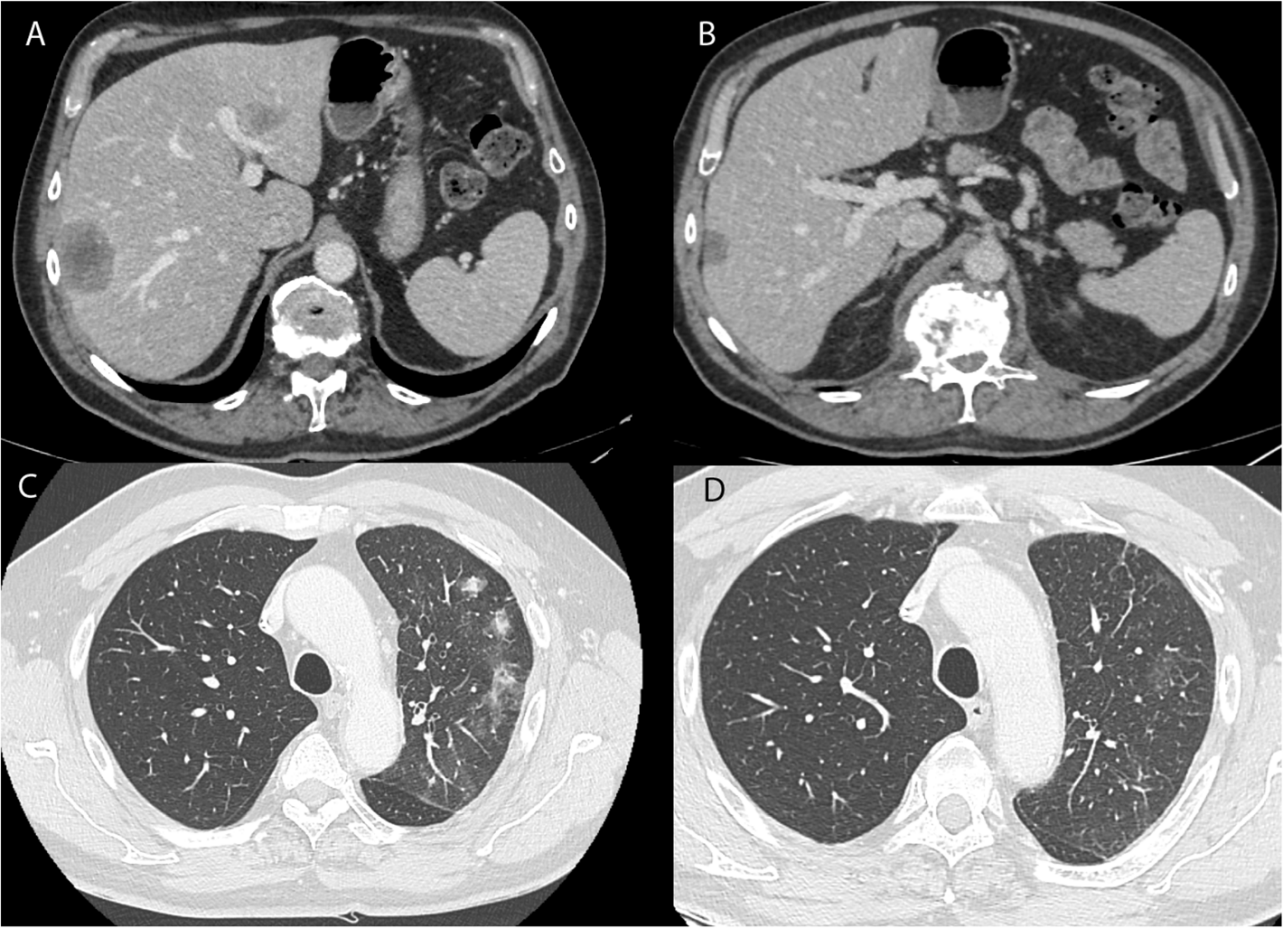
The radiological tumor evolution. **a** computed tomography (CT) scan at baseline showing hepatic metastases of the lung adenocarcinoma. **b** CT scan shows a partial response after 8 cycles of immunotherapy. **c** pulmonary progression on the CT scan at 4 months after the discontinuation of immunotherapy. **d** CT scan image showing a partial response after 8 cycles of immunotherapy rechallenge

**Fig. 3:**
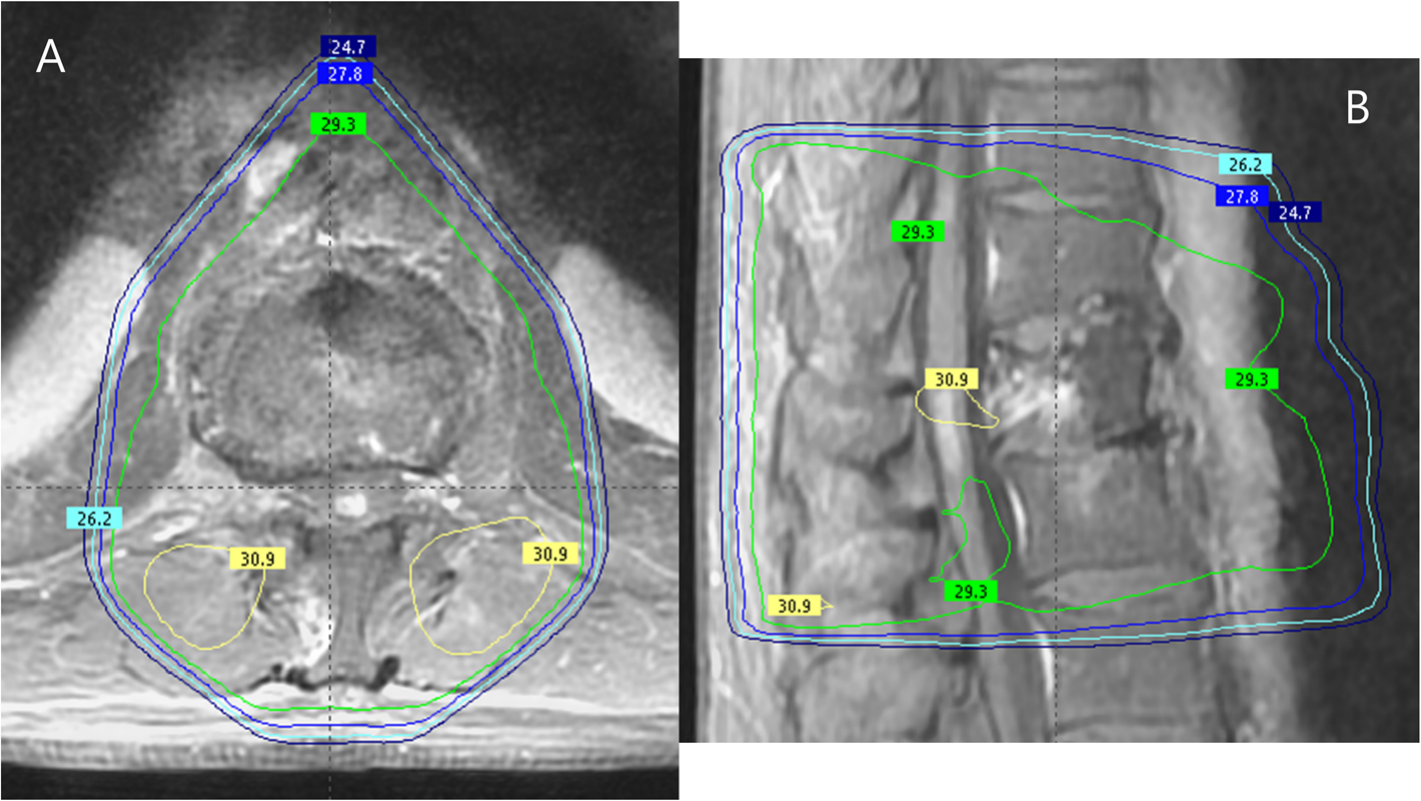
Dosimetry study for the site of myelitis*.* **a** and **b** present an image fusion between the MRI showing the location of the myelitis (gadolinium-enhanced fat-saturated T1-weighted) and the treatment plan. The angle beams used: one anterior and two oblique posterior beams. The green isodose represents 98% of the prescribed dose (29.3 Gy) and the yellow isodose 103% of the prescribed dose (30.9 Gy). On both pictures, we can see that the dose received at the site of myelitis is 30 Gy

A 68-year-old man without significant past medical history was diagnosed with advanced lung adenocarcinoma *(KRAS* mutated) with synchronous hepatic, pulmonary, and bone metastases. Because of painful L1 spinal epiduritis **(**Fig. 1a, b), without any sensory or motor deficit, tridimensional conformational radiotherapy was delivered to the vertebra T12 - L2, at a dose of 30 Gy in 10 fractions and 12 days. Fifteen days later, immunotherapy was initiated using pembrolizumab (PD-L1 expression score > 50%, no *EGFR* mutations nor *ALK* translocations). After 8 cycles (24 weeks), computed tomography (CT) evaluation showed an almost complete tumor response (Fig. 2b), but the patient began to present muscle weakness in the left lower limb, paresthesia, difficulty urinating, and rapid bowel movements. Magnetic resonance imaging (MRI) of the spine showed spinal cord edema with T1 hypointense signal and patchy gadolinium enhancement at T12-L1 levels, suggestive of focal myelitis and that the osseous tumoral involvement and epiduritis had regressed (Fig. 1c, d). As the spinal abnormalities matched the irradiated site, a dosimetric study analysis was performed, which confirmed the maximal dose of 30 Gy received in this region (Fig. 3). The cerebrospinal fluid analysis revealed moderately elevated proteinorachy (0.84 g/l). The intrathecal immunoglobulin synthesis was negative, there were no antineural antibodies, and the cytology was negative for inflammatory or tumor cells. Pembrolizumab was discontinued, and the patient received oral steroid treatment (60 mg/day), tapered over the next 2 months. After 48 h of steroid therapy, there was significant improvement of the symptomatology, which completely disappeared after 3 weeks. After 14 weeks, the patient remained asymptomatic, with radiological improvement in myelitis (Fig. 1f). Unfortunately, pulmonary disease progression was noted (Fig. 2c). In this context, pembrolizumab was resumed and, after 8 cycles, no relapse of myelitis was observed clinically nor radiologically, with partial tumor response at the CT reevaluation (Fig. 2d).

## Discussion and conclusions

In the described case, the spinal injury faithfully corresponds to the irradiated site, which strongly pleads for the influence of the previous radiotherapy in the etiology of myelitis. Moreover, the latency of appearance is in line with the one described for delayed radiation myelopathy (DRM), although at the inferior limit, and no tumoral or other possible cause was found.

However, the radiation dose received by the thoracic spine is well below the recognized tolerance dose and the doses previously reported with progressive myelopathy [4, 9]. According to previous studies, the spinal cord shows a high fractionation sensitivity, typical for late-responding normal tissues and characterized by a low ratio of the linear-quadratic parameters of the cell survival curve (α/β) [9–11]. When calculating the linear-quadratic equivalent dose at 2 Gy per fraction (LQED_2_), or the biologically equivalent dose if given in 2 Gy-fractions (for the cases when the daily fractions were higher than 2 Gy), some authors have estimated that an appropriate α/β ratio was close to 2 Gy [10], while others have favored an even lower value of 0.9 [11] or 0.87 Gy [4, 9]. Using this latter value, the estimated probability of myelopathy of the cervical spinal cord was found to be 0.03% for a total dose of 45 Gy and 0.2% for 50 Gy [9], or < 1% and < 10% for 54 Gy and 61 Gy, using conventional fractionation of 1.8–2 Gy/fraction [4]. Regarding these results, we should take into account a higher sensitivity of the cervical spinal cord than of the thoracic one [9, 11]. In accordance, earlier papers also describe a 0.4% incidence of myelopathy at 45–50 Gy [12], but some authors recommend caution when the LQED_2_ exceeds 48 Gy [10].

In our case, the patient received 30 Gy in 10 fractions and 12 days. The LQED_2_ is 37.5 Gy for α/β = 2 Gy and 40.4 Gy for α/β = 0.87 Gy. Both of these calculated values are very unlikely to cause radiation myelopathy on their own, which made us consider the existence of a predisposing factor for its occurrence.

Furthermore, the clinical course was unusual for classic radiation-induced progressive myelopathy, with a rapid, complete, and stable resolution of the symptomatology under steroid therapy.

This atypical presentation suggests the contribution of the immunotherapy by pembrolizumab to the pathogenesis in this case.

As expected, an increasing number of studies focused on the synergistic effects of radiotherapy and immunotherapy and the benefits of the combination therapy, including at the central nervous system (CNS) level [7, 8]. Although radiation necrosis is a concern after stereotactic radiotherapy for brain metastases and ICI, the majority of studies reports no significant increase of adverse effects in the setting of ICI therapy and cranial irradiation [13], and the combination therapy with palliative irradiation proves to have a tolerable safety profile [14]. Furthermore, a recent study showed that palliative stereotactic or fractionated radiotherapy for vertebral metastasis was well-tolerated and efficient in patients treated with ICI, with amelioration of the neurologic symptomatology and low-grade fatigue as the main toxicity [15].

The occurrence of myelitis after radiotherapy and ICI has been reported in only three cases at present, to our knowledge. In melanoma patients, after ipilimumab/nivolumab, with worsening after pembrolizumab [16], in another case, after treatment with ipilimumab [17] and at an NSCLC patient after durvalumab [6]. A complete recovery was not obtained in either of these cases and the incriminated drug was not rechallenged.

At a histopathological level, the changes observed in radiation-induced late spinal injuries consist of gliosis, demyelination, and areas of white matter necrosis occurring after 3–5 months of irradiation and vascular damage, as a later event, usually appearing after more than 10 months of irradiation [2, 3]. Although the cellular and molecular mechanisms are still in debate, the damage to the endothelial cells together with the oligodendrocytes seem to have major roles in the process of demyelination, with both early and late hyperpermeability and disruption of the blood – spinal cord barrier being main events in the development of spinal injury. In addition, astrocytes and microglia were also shown to have an active role in radiation myelopathy by their response to and release of inflammatory cytokines. As such, the release by these stimulated cells of TNFα has the potential to cause, directly or via IL-6, cytotoxic effects to oligodendrocytes and the endothelium, being associated with demyelination [2, 3]. Moreover, the astrocytes induce hyperpermeability through the release of VEGF and NOS [3].

A consistent description of the mechanism of toxicity at the CNS level of checkpoint inhibitors is lacking, and the rare cases are mainly reported in the presence of a CTLA-4 inhibitor [18, 19]. However, based on the observations from demyelinating inflammatory disorders, the increased migration of autoantibodies, the damage of neuronal cells by T-cells, and inflammation-mediated by cytokines, such as TNFα and IL-6, might be involved [19]. Moreover, the anti-TNFα drug, infliximab, proved successful, after the failure of steroid therapy, in the treatment of ipilimumab-induced necrotizing myelopathy [20] and of progressive transverse myelitis, which occurred after concurrent ipilimumab/nivolumab and spinal irradiation and worsened on pembrolizumab [16], both in melanoma patients.

These observations further reiterate the presumption of enhanced cytokine-mediated inflammatory reaction on focal post-radiation chronic inflammatory state as a possible CNS toxicity of the combination of radiotherapy and ICI.

Given the previously mentioned safety results of the association of radiotherapy and immunotherapy, an alternate explanation that we should take into consideration in our case is a possible radiation recall phenomenon.

Radiation recall is an acute inflammatory reaction, confined to a previously irradiated area, triggered by the administration of various chemotherapy, targeted therapies or even, recently, by immunotherapy [21].

The pathogenic mechanisms of radiation recall are not yet fully understood but a possible explanation is the hypersensitivity reaction, with the upregulation by the precipitating drug of pro-inflammatory cytokines, which are secreted at low levels by previously irradiated cells, and the exacerbation of the inflammatory reaction [21].

Radiation recall myelopathy has already been described with paclitaxel and dabrafenib [22, 23]. Likewise, several papers have reported radiation recall dermatitis or pneumonitis with the administration of ICI [24, 25].

In the existing literature referring to the radiation recall phenomenon, there have been reported good responses to steroid therapy and the rechallenging of the triggering drug does not necessarily elicit the inflammatory reaction [21], as was also the case for our patient. What is more, a safe rechallenge with nivolumab has been reported in a case of radiation recall pneumonitis [24], but never before after myelitis.

The immunotherapy could have an additional effect on the radiotherapy’s complications, amplifying the delayed inflammatory medullary reaction post-radiotherapy. However, this reaction can be reversible with the discontinuation of immunotherapy and steroid treatment and, if necessary, the rechallenge of immunotherapy remotely after the toxicity episode could remain an option, as shown by this case.

As these two treatment modalities are increasingly being used in close sequence, it is important to draw attention to the new array of potential additive adverse effects and report possible strategies for their management.

## Data Availability

Data sharing is not applicable to this article as no datasets were generated or analyzed during the current study.

## Abbreviations

ALK: Anaplastic lymphoma kinase
CNS: Central nervous system
CT: Computed tomography
CTLA-4: Cytotoxic T-lymphocyte antigen-4
DRM: Delayed radiation myelopathy
EGFR: Epidermal growth factor receptor
Gy: Gray
ICI: Immune checkpoint inhibitor
IL-6: Interleukin-6
LQED_2_: Linear-Quadratic Equivalent Dose at 2 Gy per fraction
MRI: Magnetic resonance imaging
NOS: Nitric oxide synthase
NSCLC: Non-small-cell lung cancer
PD-1: Programmed cell death protein-1
PD-L1: Programmed death-ligand 1
TNFα: Tumor necrosis factor alpha
VEGF: Vascular endothelial growth factor
